# Central and Eastern European Migrants in the United Kingdom: A Scoping Review of the Reasons for Utilisation of Transnational Healthcare

**DOI:** 10.1111/hex.14155

**Published:** 2024-07-24

**Authors:** Victoria Stepanova, Aaron Poppleton, Ruth Ponsford

**Affiliations:** ^1^ Faculty of Public Health & Policy London School of Hygiene and Tropical Medicine London UK; ^2^ School of Medicine Keele University Stoke‐on‐Trent UK

**Keywords:** communication barriers, delivery of healthcare, Eastern Europe, Eastern European people, migrants, transients, United Kingdom

## Abstract

**Background:**

An estimated 2.2 million people from Central and Eastern Europe (CEE) live in the United Kingdom. It has been documented that CEE migrants underutilise health services in the United Kingdom and, as an alternative, seek healthcare in their home country. However, reasons for seeking healthcare abroad are not always clear. This review aims to identify the reasons for the uptake of transnational healthcare among CEE migrants resident in the United Kingdom.

**Methods:**

Informed by discussions with community members, medical stakeholders and academics, a systematic scoping review was undertaken following the nine‐stage Joanna Briggs Institute framework for scoping reviews. A search strategy with MeSH terms, where relevant, was used and adapted in five academic databases, two grey literature databases and Google Scholar. Included records encompassed four concepts: migration, CEE nationalities, UK nations and healthcare utilisation, which were written in English and published between May 2004 and 2022. Data from the literature were coded, grouped and organised into themes.

**Results:**

A total of 16 publications fulfilled the inclusion criteria. There is evidence that some CEE migrants exclusively use healthcare services in the United Kingdom. However, many CEE migrants utilise healthcare both in the United Kingdom and their country of origin. Four themes were identified from the literature as to why migrants travelled to their country of origin for healthcare: cultural expectations of medical services, distrust in the UK NHS, barriers and transnational ties.

**Conclusion:**

Push factors led CEE migrants to seek healthcare in their country of origin, facilitated by ongoing transnational ties. CEE migrants frequently combine visits to their country of origin with medical appointments. Utilising healthcare in their country of origin as opposed to the United Kingdom can result in fragmented and incomplete records of medications, medical tests and surgeries and risk of unnecessary treatments and complications. This review highlights the need for more targeted health outreach with CEE groups within the United Kingdom, as well as the need for further research on the impact of national events, for example, COVID‐19 and Brexit, on transnational healthcare‐seeking behaviours.

**Patient or Public Contribution:**

The concept for this scoping review was informed by discussions with community members, medical professionals and academics, who identified it as a current issue. The results of this scoping review were discussed with healthcare stakeholders.

## 1 Introduction

The expansion of the European Union (EU) since 2004 simplified migration from countries in Central and Eastern Europe (CEE) to the United Kingdom [1]. The number of CEE nationals in the United Kingdom has fluctuated over time, influenced by the economic situation in the United Kingdom, the 2016 Brexit referendum and the conflict in Ukraine. The current population of CEE migrants in the United Kingdom is sizeable, with estimates ranging between 1.6 and 3.3 million [2]. Among this population, Poland and Romania are currently the most represented CEE nations [3].

EU citizens who were ordinarily resident (a person is ordinarily resident if they are living in the United Kingdom lawfully, voluntarily or for settled purposes [4]) in the United Kingdom before Brexit are entitled to ‘free at the point of use’ care within the UK National Health Services (NHS), the same as UK nationals [5]. Despite having access to healthcare services in the United Kingdom, it has been documented that CEE migrants frequently and voluntarily travel back to their country of origin (CoO) to access healthcare [6, 7, 8, 9]. There is evidence that CEE migrants are at a higher risk of poorer physical health outcomes, including obesity, cardiovascular disease, cancer and sexual health [9, 10, 11]. Research findings have shown that CEE migrants experience barriers in accessing care in the United Kingdom, including language, literacy and confusion surrounding the system and eligibility [7, 8, 9, 10]. Limited English language abilities impede migrants' abilities to engage with healthcare providers and contribute to their lack of understanding of the system, awareness of provisions available and accessing services [9, 11, 12, 13]. To improve the use of NHS services by CEE migrants resident in the United Kingdom, it is important to understand their reasons for choosing to go back to their CoO and if there are obstacles that can be overcome to improve their healthcare uptake and health outcomes.

The term ‘diasporic medical tourism’ has been used to describe migrants that travel back to their CoO for healthcare as distinguishable from ‘medical tourism’, which usually carries consumer and commercial connotations [14, 15]. Many accounts of medical tourism pertain to travel for invasive procedures that are cheaper abroad or not available in the host country. Common examples include dental, bariatric and cosmetic procedures undertaken for aesthetic reasons as opposed to medically necessary procedures, thereby transcending health boundaries [16, 17].

### 1.1 Implications for Health

Transnational healthcare utilisation can result in a lack of continuity in healthcare. Procedures or expectations for health record transfers vary between healthcare facilities and systems across Europe [18]. Incomplete health records affect the ability of healthcare providers to make appropriate prescribing and treatment choices, thereby increasing the risk of unintended harm to the patient [19]. Gaps in continuity in care also have potential financial cost implications for the healthcare system and the patient [20]. Furthermore, misuse or overuse of antibiotics increases the risk of antimicrobial resistance (AMR) development and spread [21, 22].

Migrants who put off treatments or healthcare visits until they are able to access services in their CoO risk worsening their health condition/s, affecting their quality of life and increasing the level of care required. Transnational healthcare utilisation can exacerbate health inequalities as not all migrants have the financial means to travel to their CoO. These migrants may also lack the financial means of going back to their home country to access healthcare and therefore have very limited means of accessing healthcare.

This review builds upon two previous systematic scoping reviews, Phung et al. [8] and Poppleton et al. [7], in which returning to CoO for healthcare and preferences for transnational healthcare as an alternative to using healthcare services in the United Kingdom were identified themes. A further systematic narrative literature review from 2020 on transnational social networks found that migrants pursued hybrid health‐seeking strategies, with transnational networks shaping healthcare decisions [23]. However, this review did not include literature on CEE migrants. A further review of the factors driving CEE migrants to seek transnational healthcare and how these may be shaped by transnational ties is needed to better understand these processes and identify ways of improving the utilisation of healthcare in host countries to improve continuity of care and health outcomes of CEE migrants.

This is the first review to consider the factors that influence the utilisation of diagnostic and health improvement services in their CoO by CEE migrants resident in the United Kingdom, over the NHS. The review focuses on CEE migrants' experiences of accessing NHS services and their motivations for receiving care in their CoO aiming to inform service providers, policymakers, charity and health stakeholders on reasonable adjustments to improve the utilisation of NHS services by CEE migrants in the United Kingdom. Conversations with community members, medical professionals and academics in the United Kingdom who work with migrants shaped the concept for this review and the interpretation of its findings.

A systematic scoping review was undertaken due to the broad and exploratory nature of the topic. This approach allowed for the identification and inclusion of heterogeneous literature and the construction of an overview of the different concepts that contribute to an understanding of transnational healthcare usage among CEE migrants in the United Kingdom.

Scoping reviews are used to identify and provide an overview of the available evidence for a specific field, irrespective of study quality and not limited to a specific source [24, 25]. Compared to a systematic review, scoping reviews employ broader questions as they aim to summarise the breadth of evidence with less restrictive inclusion criteria [26, 27]. This can identify literature that otherwise may be overlooked, specific characteristics related to a concept and research gaps [26], which can benefit policymakers and stakeholders.

## 2 Methods

### 2.1 Patient and Public Contribution

The concept and interpretation of findings for this scoping review were informed by discussions with community members, medical professionals and academics. These individuals identified the review topic as a current issue and shared their personal experiences of transnational healthcare utilisation.

### 2.2 Review Structure

This review was guided by the nine‐stage framework proposed by the Joanna Briggs Institute (JBI) [28, 29], which is informed by the work of Arksey and O'Malley [25] (Supporting Information S1: Appendix 1). The JBI framework provides a clear and structured process for conducting a scoping review. The framework is aligned with the Preferred Reporting Items for Systematic Review and Meta‐Analyses extension for Scoping Reviews (PRISMA‐ScR), a checklist with 20 essential reporting items when completing a scoping review [30]. This review adheres to the PRISMA‐ScR (Supporting Information S1: Appendix 2). Using both a JBI framework and PRISMA‐ScR ensures adherence to standardised procedures for conducting and reporting a scoping review [29].

The nine stages consist of the following:

1. Defining and aligning the objective/s and question/s.
2. Developing and aligning the inclusion criteria with the objective/s and question/s.
3. Describing the planned approach to evidence searching, selection, data extraction and presentation of the evidence.
4. Searching for the evidence.
5. Selecting the evidence.
6. Extracting the evidence.
7. Analysis of the evidence.
8. Presentation of the results.
9. Summarising the evidence in relation to the purpose of the review, making conclusions and noting any implications of the findings.

### 2.3 Eligibility Criteria for Included Articles

The inclusion criteria were informed by the PCC (Population, Concept and Context) framework (Table 1) [31] as follows:

→ Population—Documented migrants from EU2 or EU8 countries resident in the United Kingdom.
→ Concept—Travelling back to CoO to utilise health services or have the desire or intention to utilise healthcare in CoO.
→ Context—CEE migrants living in any of the four devolved UK nations.

**Table 1:** Inclusion/exclusion criteria.

Criteria	Inclusion	Exclusion
Population	CEE documented migrants from any of the EU2 or EU8 countries; not restricted to any age or sex	Asylum seekers, refugees, transients and undocumented migrants
Concept	Studies must include information about CEE desire/intention for uptake of healthcare in their country of origin (any of the EU2 or EU8 countries) or travelling to their home country to utilise health services	
Context	CEE migrants settled or living in any of the four UK nations	CEE migrants not resident in the United Kingdom
Types of healthcare	Preventative care Primary, secondary and tertiary	Dental care, cosmetic surgery, conception/fertility services, mental health and bariatric surgery
Study design	All study designs	None
Publication type	Primary research; grey literature	Reviews
Timeframe	Literature published from 1 May 2004	Literature published before 1 May 2004
Language	English or Russian	Literature in any languages other than English and Russian

This review focused on CEE migrants being able to or having the option to freely travel back to their CoO. The literature surrounding the concepts of asylum seekers, refugees, transients and undocumented migrants was not included because these groups of people are:

- forcibly displaced
- fleeing danger or persecution and are thus not in a position to travel back to their CoO
- may lack proper documentation to be able to travel across borders or the risk to do so is too high.

The literature had to focus on healthcare or health‐related practices and include findings relevant to healthcare utilisation in the migrant's CoO. Relevant literature where transnational healthcare was not the primary focus was still included.

Literature had to pertain to healthcare utilisation in relation to preventative health or primary, secondary or tertiary healthcare. Literature that focused on cosmetic, dental or bariatric treatment was excluded due to such procedures potentially being classified as ‘aesthetic medicine’, in which these procedures are not medically necessary [32]. Although bariatric surgery can be undertaken by the NHS where deemed medically necessary, numbers are limited by strict and specific criteria. Additionally, although oral health is a key indicator of overall health [33], only specific groups are eligible for free NHS treatment throughout the United Kingdom with some variability within the four nations [34, 35, 36, 37].

Literature was included if it contained primary evidence. Reviews and evidence syntheses were not included.

The chosen timeframe was intended to capture literature since the accession of the EU2 and EU8 countries into the EU, which allowed for freedom of movement. Despite the United Kingdom's departure from the EU in 2020, resident EU citizens are allowed to remain in the United Kingdom under the EU settlement scheme [38].

Finally, literature was included if written in English or Russian as the primary author is fluent in Russian. Literature in any other language was excluded due to resource limitations.

The experience of migrants seeking healthcare in their adopted country was discussed with stakeholders to get a broad understanding of the issues. The primary author has lived family experience of migrating to a country that is culturally different to their birth country, which necessitated the learning of a new language and transgressing cultural barriers.

### 2.4 Search Strategy (Searching for the Evidence)

The search strategy was drafted by the primary author and refined after consultation with a university information scientist. PCC was used to guide the development of the search strategy [26, 28]. Search terms aimed to capture four concepts related to migration, CEE nationalities, UK nations and healthcare utilisation. MeSH terms were used (where available) and adapted to each database.

Five academic databases (Embase, CINAHL, MEDLINE, Scopus and Web of Science), two grey literature databases (Global Health and Social Policy and Practice) and the first 10 pages of Google Scholar were searched (Supporting Information S1: Appendix 3). The reference lists of all included studies were hand‐searched. All databases were searched on 15 July 2022.

### 2.5 Article Selection (Selecting the Evidence)

Duplicates were removed, and the remaining articles were screened on title and abstract using the inclusion/exclusion criteria as described earlier. The full text of the remaining reports was screened to identify a final set of relevant articles.

### 2.6 Data Extraction (Extracting the Evidence)

The primary author developed a data extraction template on Microsoft Excel, as guided by JBI, and extracted data on author, year, title, publication type, CEE nationality/population, UK location, years spent in the United Kingdom, main themes and subthemes from each article.

### 2.7 Data Synthesis (Analysis of the Evidence)

Given the inclusion of quantitative, qualitative and mixed‐methods studies, JBI guidelines for mixed‐methods reviews guided data synthesis [39]. The convergent integrated approach was used on the basis that both quantitative and qualitative data can provide useful insights to address the research question. This involved the transformation of quantitative data to qualitative (‘qualitizing’) through narrative interpretation of quantitative results [40], which allowed for the integration of the data in an inductive thematic synthesis approach [41, 42, 43]. The data from the literature were coded, grouped and organised into themes.

### 2.8 Quality Appraisal (Analysis of the Evidence)

Quality appraisal is not required for scoping reviews [25, 31] but was undertaken in this review to aid interpretation of review findings. Qualitative studies were critically appraised using the Critical Appraisal Skills Programme (CASP) Checklist for qualitative studies [44]. Mixed‐methods and quantitative studies were appraised using the Mixed Methods Appraisal Tool (MMAT) [45]. No critical appraisal tool could be identified for the policy report [46].

## 3 Results

### 3.1 Study Selection and Process

Nine hundred and thirty‐nine results were identified from eight databases, and four additional results were identified through hand‐searching reference lists of relevant studies. Following automatic and manual deduplication, 457 records were imported into Rayyan [47] to carry out the screening process.

Three hundred and seventy‐four results were excluded on the basis of title and abstract. The remaining 83 results were read in full text. After full‐text screening, 16 records were identified as eligible for inclusion (Supporting Information S1: Appendix 5). Reference lists of included articles were screened with no further results identified.

**Figure 1:**
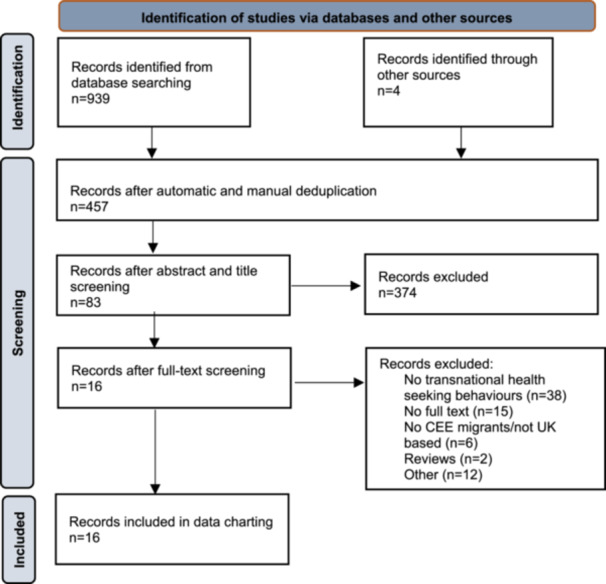
PRISMA flow diagram.

Figure 1 of our PRISMA flowchart illustrates the review process. Sixteen publications were identified as eligible for inclusion.

### 3.2 Study Characteristics

The included literature was published between 2011 and 2022, with half (*n* = 8) published between 2020 and 2022. Twelve reports were qualitative studies, two were mixed methods, one was quantitative and one was a policy report based on qualitative research. All literature was in English. The majority focused on Polish migrants (*n* = 15), with Romanians being the next most frequently described (Table 2). Publications focused on CEE migrants in England (*n* = 7), Scotland (*n* = 5) or the United Kingdom more broadly (*n* = 4). Notably, there was no specific mention of experiences of CEE migrants within Wales or Northern Ireland and no publications, which included Estonian or Slovenian migrants.

**Table 2:** Nationality coverage.

CEE group	No. of publications
Polish	15
Romanian	6
Slovakian	4
Czech	3
Hungarian	3
Bulgarian	2
Lithuanian	2
Latvian	1
Estonian, Slovenian	0

### 3.3 Quality of the Evidence

The CASP‐recommended classification of quality based on high, moderate or low was used in this review [48]. These were determined based on the scores in percentage, consisting of the number of questions that met the criterion ‘yes’ divided by the total number of applicable questions. Studies that scored below 50% were classified as low quality, studies between 50% and 79% were moderate and studies that scored 80% and above were high quality. All studies included in this review were deemed to be of high quality, despite some methodological limitations. No appraisal was done for the policy report as there is no suitable checklist. No studies were excluded on the basis of their quality, and there was no weighting in the evidence synthesis.

### 3.4 Evidence Synthesis

Findings were grouped into two broad overarching categories pertaining to (1) CEE migrants who use health services exclusively in the United Kingdom and (2) CEE migrants who combine NHS services with healthcare utilisation in their CoO. Although the review did not initially set out to also explore perceptions of CEE migrants using only health services in the United Kingdom, some of the literature included findings related to this group, which have been included in the review as they provide evidence of varied experiences and practices among CEE migrants. The second category of CEE migrants combining NHS services with healthcare services in their CoO was split into four separate themes that explained the health‐seeking behaviour of those combining utilisation of the NHS and healthcare in their CoO: cultural expectations of medical services, trust/distrust, barriers and transnational ties. The coding structure is presented in Supporting Information S1: Appendix 4.

#### 3.4.1 Using Health Services Exclusively in the United Kingdom

Although most CEE migrants within the 16 publications utilised healthcare in their CoO either exclusively or in addition to the NHS, three studies revealed that some CEE migrants used healthcare exclusively in the United Kingdom [49, 50, 51]. Reasons included convenience and cheaper cost. Financial considerations were a major factor, as certain migrants could not afford the costs associated with utilising healthcare in their CoO, which usually consisted of purchasing airfare and health services abroad. Moreh, McGhee, and Vlachantoni [49] described migrants who solely used NHS healthcare had made this decision as part of their perceived identity as United Kingdom residents.

Two studies contained evidence that Romanians had less trust in the Romanian healthcare system and felt the NHS was more trustworthy in terms of quality [49, 51]. Expectations of bribes and gratuity in Eastern European national healthcare systems were another deterrent for travelling back to individuals' CoO for healthcare.

#### 3.4.2 Using Healthcare Services in the United Kingdom and CoO

The majority of studies illuminated that CEE migrants utilised healthcare both within the NHS and in their CoO. The following themes illustrate the factors shaping CEE migrants' desire to seek healthcare in their CoO.

##### 3.4.2.1 Cultural Expectations of Medical Services

Mismatched cultural expectations of healthcare services provided in the United Kingdom were the most common theme [49, 50, 52, 53, 54, 55, 56, 57, 58, 59, 60, 61, 62, 63]. CEE migrants were frustrated with the general practitioner (GP) acting as the gatekeeper, with no direct or easy access to specialists in the NHS compared to their CoO. Women were surprised when cervical cancer screening was carried out by nurses and not by gynaecologists [51, 60]. In one study of Polish migrants, longer intervals between screenings, both breast and cervical, and a difference in age eligibility were also unexpected, compared to the guidelines in Poland, and were thought to be cost‐saving measures [54]. More frequent screenings, which usually included a general check‐up, were a motivating factor for CEE migrants to travel to their CoO [54, 57, 60].

Migrants were also put off using NHS services due to waiting times [52, 53, 55, 58] and found it easier and more efficient to access specialist care in their CoO. A widespread complaint about GPs was the perceived over‐reliance on paracetamol and reluctance to prescribe medications, such as antibiotics [49, 50, 51, 52, 58, 62]. The research carried out by Healthwatch Reading [62] revealed that participants labelled their GPs as ‘the paracetamol service’. Individuals also identified discrepancies in diagnoses between the United Kingdom and their CoO [59, 61]. One case study by Troccoli et al. [61] illustrated the way a Polish woman navigated the healthcare systems in the United Kingdom and Poland, with her son being diagnosed with asthma in Poland before he was diagnosed in the United Kingdom, due to variation in diagnostic criteria. She also reflected on getting blood tests done both in Poland and in the United Kingdom because of ‘differences in what hormone levels are considered pathological in the two countries’ (p. 2011). CEE migrants often utilised private testing in their CoO to gain access to specialist care or medication within the NHS, which they felt was otherwise difficult to obtain through their GP [52, 54, 58, 61, 62].

Lack of immediate access to test results in the United Kingdom was another source of frustration [54, 59, 61], as evidenced by one participant in the study: ‘Getting test results is different. In Poland you can get them in your hands while here you cannot see them at all’ [51]. This further contributed to CEE migrants' preference to seek transnational healthcare.

However, although longer waiting times and lack of direct access to hospital specialists were often seen as push factors, one study revealed that these were often preferred over expectations for gratuities or bribes in their CoO [61].

##### 3.4.2.2 Trust/Distrust of Healthcare in the United Kingdom

Although CEE migrants utilised NHS healthcare, they often returned to their CoO to seek reassurance, to obtain second opinions of the tests done in the United Kingdom or to compare advice offered by GPs in the United Kingdom [51, 52, 54, 59, 61]. Some studies indicated that CEE migrants had little confidence in their GPs, with some evidence showing this stemmed from their perception that GPs ‘looked at photos on the internet’ [58] and ‘typed away on the computer’ [53] to diagnose and prescribe. There was also scepticism regarding the expertise and qualifications of GPs and nurses, with the view that some of the services they provided should have been undertaken by specialists, such as vaccine administration and smear tests [53, 54, 60, 63], which was largely the case in the CoO.

In contrast to their feelings about GPs, there is evidence that CEE migrants had positive experiences with hospital care. CEE migrants largely appreciated the patient‐centred approach they received in the United Kingdom, compared to what they felt were pushy and paternalistic styles in their CoO [50, 54, 55, 56, 58], whereas one study revealed that Polish migrants viewed the patient‐centred approach as a sign of incompetence [52].

##### 3.4.2.3 Barriers to Accessing Healthcare in the United Kingdom

Language, written and spoken, and a lack of knowledge about the healthcare system in the United Kingdom were frequently cited barriers to accessing healthcare [49, 52, 53, 54, 60, 62, 63]. Although in most cases individuals wanted a translator or interpreter to assist with appointments and were frustrated with the lack of assistance, one study revealed how people specifically sought GPs who spoke their language because they did not want an interpreter, due to perceived ‘awkwardness/embarrassment’ in intimate situations [54]. CEE migrants feared or could not afford to take time off work to attend health appointments due to a loss of income [54]. Many migrants saw it as more cost‐effective or easier to schedule healthcare appointments for their leisurely visits ‘back home’, in their CoO, preventing the need for further leave from work [51, 52, 54].

##### 3.4.2.4 Transnational Ties

Half of the included articles (8/16) revealed that CEE migrants frequently combined seeking healthcare in their CoO with travel for holidays and to visit relatives and friends, while sometimes taking care of ‘non–health‐related matters’ at the same time [61]. During such visits, migrants often took the opportunity to visit a doctor or see other healthcare professionals, which were easier and quicker to get access compared to the United Kingdom [51, 52, 53, 54, 55, 58, 59, 62]. Referring to Poland, one migrant said ‘I go at least once a year, my dad makes me an appointment with a nephrologist and a gynaecologist’ [52]. Some also took the opportunity to stock up on medications that were either not available in the United Kingdom or were not easily accessible and required prescriptions, such as antibiotics [52, 55, 56, 58, 61]. Some CEE migrants also wanted to maintain registration and communication with doctors in their home countries, due to their uncertainty of long‐term settlement in the United Kingdom [51, 58]. The availability of family convalescent care also influenced individual's decisions to seek healthcare in the CoO [61]. Two studies described CEE migrants telephoning relatives or healthcare professionals in the CoO from the United Kingdom to seek medical advice or second opinions [58, 63].

## 4 Discussion

### 4.1 Summary of Key Findings

This review synthesises the evidence on the influences and motivations of CEE migrants living in the United Kingdom to utilise healthcare in their CoO. We identify CEE migrants' unmet cultural expectations of medical services, level of trust/distrust in NHS services, barriers to NHS service use and maintenance of transnational ties as key factors influencing the ongoing utilisation of transnational healthcare.

Studies included in this review suggested that many CEE migrants utilise healthcare in their CoO either instead of or in addition to utilising NHS services. Reasons for the utilisation of transnational healthcare were largely consistent across the CEE nationalities represented in this review. Two studies included findings that some CEE migrants preferred to utilise healthcare solely in the United Kingdom as opposed to their CoO, which was attributed to the levels of distrust with the doctors ‘at home’ and the expectations of bribes or gratuity [49, 51]. Keeping with Moreh, McGhee, and Vlachantoni [49], we identified that healthcare utilisation in the United Kingdom was associated with a sense of belonging—through living and paying taxes in the United Kingdom. Despite this, fundamental differences in expectations of health services in the United Kingdom, such as differing prescribing practices, especially for antibiotics, contributed to distrust of the NHS. These differences in practice may be due to the emphasis on antibiotic stewardship in the United Kingdom, with greater clinician adherence to national guidelines and thus restrictive prescribing practice to curb AMR [64, 65]. CEE countries have higher rates of AMR [66], and studies have shown that countries such as Poland, Romania and Czechia have more liberal prescribing tendencies [67, 68, 69]. Findings reported differences in diagnosis and treatment between nations, which may be due to different thresholds or different treatment practices, complicating transferability of healthcare.

Studies showed that transnational ties facilitated the decision to seek healthcare in CEE's CoO. CEE migrants are maintaining links with their CoO, both with family members and health networks. Medical appointments are incorporated with visits back home. These social networks also shape CEE migrants' health‐related practices by providing information and advising, both in person and on the phone [58, 63]. However, these connections are dynamic and can change over time, which can influence the health‐seeking decisions of migrants and the way they utilise health resources [58].

UK–resident CEE migrants utilising healthcare in their CoO are large purchasers of private healthcare. Although this involves financial transactions and patients becoming customers, this differs from medical tourism. CEE migrants are travelling to familiar locations and are nationals with personal connections, rather than tourists. Utilising healthcare in two different countries, or in a country other than where CEE migrants are resident, can have implications for their continuity of care. These patterns of healthcare utilisation also raise questions about whether the onus is on the NHS to provide continuity of care for migrants voluntarily returning to their CoO to undergo surgical procedures.

Migrants underutilising healthcare in the United Kingdom may be delaying treatment until scheduled travel to their CoO. This can exacerbate health conditions that can lead to them requiring additional or more complicated care in the long run [22]. This review identified CEE migrants' frustration with NHS waiting times. It would be noteworthy to consider how the COVID‐19 pandemic and its associated impact on NHS provision have influenced CEEs' perceptions of the NHS and transnational healthcare. Additionally, it would be valuable to understand how travel restrictions during the COVID‐19 pandemic impacted CEE migrants' abilities, routes and decisions to seek healthcare in their CoO.

Although some studies included information on CEE participants' length of residency/years spent in the United Kingdom [51, 52, 53, 54, 56, 57, 59, 62, 63], most made no explicit connection or analysis of what impact this had or may have had on the uptake of health services in the United Kingdom and CoO. Many of the participants in the studies had lived in the United Kingdom for several years. It has been posited that integration tends to improve with the length of residence [70, 71], but it is not currently known if greater integration and longer residency have any effect on the use of NHS services and transnational healthcare.

The literature included in this review involved participants, which helps provide a deeper understanding of their experience with the healthcare system in the United Kingdom, and how patient perspectives can strengthen findings and the way research is taken up in practice.

### 4.2 Implications for Policy and Research

CEE's decision to use transnational healthcare stems from fundamental beliefs and expectations about healthcare. Factors such as NHS prescribing practices and duration of waiting times for specialist care are structural factors affecting all communities in the United Kingdom. However, steps can be taken to support and increase CEE migrants' use of NHS services and to reduce the risk of potentially harmful consequences in utilising transnational healthcare. In 2021, Poland and Romania were the first and fifth, respectively, most common nationalities in the United Kingdom [3]. Measures to increase the confidence and trust of these nationals in the NHS would support wider CEE migrant engagement with the United Kingdom.

Currently, the NHS has a significant backlog of care [72]. In the short term, transnational healthcare utilisation by CEE migrants has the potential to reduce demand on the NHS. However, a reliance on transnational healthcare risks greater long‐term challenges for the NHS. Transnational healthcare utilisation can contribute to and exacerbate informational discontinuity through gaps in availability and recording of health information [73]. CEEs' health needs will likely increase, and their ability to travel decreases with age. Potentially unmet or inadequately met health needs risk inequity, particularly in individuals with multiple comorbidities, complex care needs or limited capacity. Targeted outreach towards CEE migrants could encourage the uptake of healthcare services in the United Kingdom and facilitate the sharing of health records, ensuring comprehensive care.

Steps can be taken to overcome barriers to CEE engagement with the NHS. In the short term, facilitating post–Brexit work permit/visa requirements could support the recruitment of staff with knowledge of CEE languages [74]. The number, access and range of digital and print resources in CEE languages could be widened, with greater use of co‐design. A single central access point would support standardisation, increase quality and reduce potential for confusion in accessing care, particularly for common ailments, which offers information and clear advice on accessing care through the NHS would help CEE migrants to find the correct route for healthcare and increase their understanding of what the NHS can offer.

These provisions should be underpinned by improvements in data collection. At the point of healthcare delivery, CEE migrants are usually categorised in the NHS as ‘White—Any other White background’ [75] with no further recording of ethnicity, culture or language differences. This precludes the monitoring needs of this population in healthcare consultations.

This review was also conducted at a snapshot in time and does not capture the most recent challenges faced by CEEs living in the United Kingdom. As of 2021, EU citizens moving to the United Kingdom are required to pay the immigration health surcharge to use NHS services [76]. It is unclear whether or how the surcharge will influence CEE migrants' engagement with NHS services and their decision to seek healthcare in their CoO. The COVID‐19 pandemic led to travel restrictions and changes in NHS care delivery. Their impact on CEEs' health and utilisation of NHS and transnational healthcare requires further exploration. The number of Ukrainians in the United Kingdom has increased significantly since 2022 [77]. Given the cultural and linguistic similarities with some EU8 and EU2 countries [78, 79], and emerging reports of transnational healthcare usage by Ukrainians in the United Kingdom [80], findings from a review of CEE health may be of direct relevance to this community.

### 4.3 Strengths and Limitations

This scoping review synthesises the available literature on healthcare utilisation of CEE migrants living in the United Kingdom. Strengths of this review include a systematic and comprehensive search using eight databases. The findings are consistent with previous reviews in that CEE migrants utilise transnational healthcare, either in conjunction with or as a replacement to the NHS, due to their expectations and experiences of services in the NHS. This review demonstrates CEE migrants' experiences and drivers for utilising healthcare in their CoO and adds that maintaining transnational ties plays a role in these decisions.

By using a scoping review methodology, a set number of databases were searched, which may have resulted in missing relevant studies. Fifteen articles were inaccessible, which means that some potentially relevant publications were excluded. Given no identified published research specifically described CEEs' experience in Wales or Northern Ireland, it is unclear whether the review findings are applicable to the devolved NHS care in these localities. This review focused on transnational healthcare use by CEE migrants rather than their use of healthcare in the United Kingdom, whereas some of the included literature had a focus on general healthcare usage in the United Kingdom. Focusing on transnational healthcare utilisation by CEE migrants does not represent the experiences of CEE migrants who utilise the NHS or private healthcare in the United Kingdom.

We endeavoured to involve community members in the review process. Further research should seek to facilitate full participant engagement at all stages to ensure that results are relevant to the people being reported on.

## 5 Conclusion

This scoping review demonstrates that CEE migrants' unmet cultural expectations of medical services, trust/distrust of the NHS, barriers to NHS service use and transnational ties influence their ongoing utilisation of transnational healthcare. These push factors lead many CEE migrants in the United Kingdom to seek healthcare in their CoO, facilitated by ongoing personal transnational ties, either instead of or in addition to utilising NHS services. This duality risks fragmented care and health inequity. Improved data collection on service use and resources for navigating the NHS could improve understanding and access to the NHS services for CEE migrants in the United Kingdom. Further research is required to explore how Brexit, the COVID‐19 pandemic and the conflict in Ukraine have influenced CEEs' healthcare utilisation in the United Kingdom and transnationally.

## Author Contributions

**Victoria Stepanova:** conceptualisation, writing – original draft, writing – review and editing, resources, formal analysis, methodology, data curation, investigation, visualisation, project administration. **Aaron Poppleton:** writing – original draft, writing – review and editing, resources, conceptualisation, supervision, project administration, methodology, visualisation. **Ruth Ponsford:** funding acquisition, writing – review and editing, writing – original draft, supervision, conceptualisation, resources, project administration, visualisation, methodology.

## Conflicts of Interest

The authors declare no conflicts of interest.

## Supporting information

Supporting information.

## Data Availability

Data sharing is not applicable to this article as no data sets were generated or analysed during the current study.
