# Supplementary material for: Central and Eastern European Migrants in the United Kingdom: A Scoping Review of the Reasons for Utilisation of Transnational Healthcare
Source: Health Expect. 2024 Jul 24;27(4):e14155. doi: 10.1111/hex.14155 (PMC11266902; doi:10.1111/hex.14155)
Supplement: Supplementary file 1 — Supporting information. [file HEX-27-e14155-s001.docx]

**Appendices**

**Appendix 1: The scoping review framework**

Scoping Review Frameworks, table from Joanna Briggs Institute (35)

| **Arksey and O’Malley framework** | **Enhancements proposed by Levac et al** | **Enhancements proposed by Peters et al** |
| --- | --- | --- |
| Identifying the research question | Clarifying and linking the purpose and research question | Defining and aligning the objective/s and question/s |
| Identifying relevant studies | Balancing feasibility with breadth and comprehensiveness of the scoping process | Developing and aligning the inclusion criteria with the objective/s and question/s |
| Study selection | Using an iterative team approach to selecting studies and extracting data | Describing the planned approach to evidence searching, selection, data extraction, and presentation of the evidence |
| Charting the data | Incorporating a numerical summary and qualitative thematic analysis | Searching for the evidence |
| Collating, summarizing, and reporting the results | Identifying the implications of the study findings for policy, practice, or research | Selecting the evidence |
| Consultation (optional) | Adopting consultation as a required component of scoping study methodology | Extracting the evidence |
|  |  | Analysis of the evidence |
|  |  | Presentation of the results |
|  |  | Summarizing the evidence in relation to the purpose of the review, making conclusions and noting any implications of the findings |

**Appendix 2: Prisma-ScR Checklist**

| **SECTION** | **ITEM** | **PRISMA-ScR CHECKLIST ITEM** | **REPORTED ON PAGE #** |
| --- | --- | --- | --- |
| **TITLE** | | | |
| Title | 1 | Identify the report as a scoping review. | 1 |
| **ABSTRACT** | | | |
| Structured summary | 2 | Provide a structured summary that includes (as applicable): background, objectives, eligibility criteria, sources of evidence, charting methods, results, and conclusions that relate to the review questions and objectives. | 3-5 |
| **INTRODUCTION** | | | |
| Rationale | 3 | Describe the rationale for the review in the context of what is already known. Explain why the review questions/objectives lend themselves to a scoping review approach. | 4-5 |
| Objectives | 4 | Provide an explicit statement of the questions and objectives being addressed with reference to their key elements (e.g., population or participants, concepts, and context) or other relevant key elements used to conceptualize the review questions and/or objectives. | 7-8 |
| **METHODS** | | | |
| Protocol and registration | 5 | Indicate whether a review protocol exists; state if and where it can be accessed (e.g., a Web address); and if available, provide registration information, including the registration number. | n/a |
| Eligibility criteria | 6 | Specify characteristics of the sources of evidence used as eligibility criteria (e.g., years considered, language, and publication status), and provide a rationale. | 8 |
| Information sources* | 7 | Describe all information sources in the search (e.g., databases with dates of coverage and contact with authors to identify additional sources), as well as the date the most recent search was executed. | 8 |
| Search | 8 | Present the full electronic search strategy for at least 1 database, including any limits used, such that it could be repeated. | Appendix 3 |
| Selection of sources of evidence† | 9 | State the process for selecting sources of evidence (i.e., screening and eligibility) included in the scoping review. | 8-9, 10 |
| Data charting process‡ | 10 | Describe the methods of charting data from the included sources of evidence (e.g., calibrated forms or forms that have been tested by the team before their use, and whether data charting was done independently or in duplicate) and any processes for obtaining and confirming data from investigators. | 9 |
| Data items | 11 | List and define all variables for which data were sought and any assumptions and simplifications made. | Appendix 4 |
| Critical appraisal of individual sources of evidence§ | 12 | If done, provide a rationale for conducting a critical appraisal of included sources of evidence; describe the methods used and how this information was used in any data synthesis (if appropriate). | 9 |
| Synthesis of results | 13 | Describe the methods of handling and summarizing the data that were charted. | 9 |
| **RESULTS** | | | |
| Selection of sources of evidence | 14 | Give numbers of sources of evidence screened, assessed for eligibility, and included in the review, with reasons for exclusions at each stage, ideally using a flow diagram. | 10 |
| Characteristics of sources of evidence | 15 | For each source of evidence, present characteristics for which data were charted and provide the citations. | 11 |
| Critical appraisal within sources of evidence | 16 | If done, present data on critical appraisal of included sources of evidence (see item 12). | 12 |
| Results of individual sources of evidence | 17 | For each included source of evidence, present the relevant data that were charted that relate to the review questions and objectives. | Appendix 5 |
| Synthesis of results | 18 | Summarize and/or present the charting results as they relate to the review questions and objectives. | 12-15 |
| **DISCUSSION** | | | |
| Summary of evidence | 19 | Summarize the main results (including an overview of concepts, themes, and types of evidence available), link to the review questions and objectives, and consider the relevance to key groups. | 16-17 |
| Limitations | 20 | Discuss the limitations of the scoping review process. | 19 |
| Conclusions | 21 | Provide a general interpretation of the results with respect to the review questions and objectives, as well as potential implications and/or next steps. | 20 |
| **FUNDING** | | | |
| Funding | 22 | Describe sources of funding for the included sources of evidence, as well as sources of funding for the scoping review. Describe the role of the funders of the scoping review. | n/a |

**Appendix 3: Database Searches**

| Medline | |
| --- | --- |
| 1 | "emigrants and immigrants"/ or "transients and migrants"/ or “emigration and immigration” |
| 2 | (migra* or emigra* or immigra* or foreig* or diaspor |
| 3 | 1 or 2 |
| 4 | europe, eastern/ or baltic states/ or estonia/ or latvia/ or lithuania/ or bulgaria/ or czech republic/ or hungary/ or poland/ or romania/ or slovakia/ or slovenia/ or Croatia |
| 5 | (Eastern Europ* or East Europ* or Central Europ* or Czech Republic or Czec* or Hungar* or Poland or Polish or Pole or Poles or Polska or Slovaki* or Sloveni* or Estoni* or Latvi* or Lithuani* or Roma* or Roma or Baltic State* or Bulgari* or Croati*) |
| 6 | 4 or 5 |
| 7 | united kingdom/ or england/ or northern ireland/ or scotland/ or wales/ or England/ or London |
| 8 | (United Kingdom or England or Northern Ireland or Scotland or Wales or England or London or Britain) |
| 9 | 7 or 8 |
| 10 | "delivery of health care"/ or culturally competent care/ or "delivery of health care, integrated"/ or health services accessibility/ or medical tourism/ or primary health care/ or secondary care/ or tertiary healthcare/ or health knowledge, attitudes, practice/ or health facilities/ or health services/ or attitude to health |
| 11 | (healthcare or "health-care" or health servic* or medica* or NHS or "national health servic*" or care or health belie* or "health-seeking") |
| 12 | (health adj2 facilit*) |
| 13 | (attitude adj3 health). |
| 14 | (health adj2 behavio*). |
| 15 | 10 or 11 or 12 or 13 or 14 |
| 16 | 3 and 6 and 9 and 15 |
| 17 | Limit 16 to yr=”2004-2022” |

| CINAHL | |
| --- | --- |
| S18 | S3 AND S6 AND S9 AND S17 |
| S17 | S10 OR S11 OR S12 OR S13 OR S14 OR S15 OR S16 |
| S16 | attitude N3 health |
| S15 | health N2 behavio* |
| S14 | "health-care" or healthcare or "health servic*" or medica* or NHS or "national health servic" or care OR "health belie*" or "health facilit*" |
| S13 | (MH "Health Beliefs") OR (MH "Attitude to Health") |
| S12 | (MH "Medical Care") |
| S11 | (MH "Health Services") OR (MH "Health Facilities") |
| S10 | (MH "Tertiary Health Care") OR (MH "Health Care Delivery") OR (MH "Health Care Delivery, Integrated") OR (MH "Health Services Accessibility") OR (MH "Medical Tourism") OR (MH "Primary Health Care") OR (MH "Secondary Health Care") |
| S9 | S7 OR S8 |
| S8 | "United Kingdom" or UK or Britain or England or London or "Northern Ireland" or Wales or Scotland |
| S7 | (MH "United Kingdom") OR (MH "Great Britain") OR (MH "Northern Ireland") OR (MH "Wales") OR (MH "Scotland") OR (MH "England") |
| S6 | S4 OR S5 |
| S5 | "Eastern Europ*" or "East Europ*" OR "Central Europ*" OR "Baltic Stat*" OR Bulga* OR Czech* OR "Czech Republic" OR Croat* OR Hungar* OR Poland OR Pole OR Poles OR Polska OR Roma* OR Roma OR Slova* OR Slove* OR Estoni* OR Latvi* OR Lithuani* |
| S4 | (MH "Europe, Eastern") OR (MH "Baltic States+") OR (MH "Bulgaria") OR (MH "Croatia") OR (MH "Czech Republic") OR (MH "Hungary") OR (MH "Romania") OR (MH "Poland") OR (MH "Slovakia") OR (MH "Slovenia") |
| S3 | S1 OR S2 |
| S2 | migra* or emigra* or immigra* or foreig* or diaspora* |
| S1 | (MH "Emigration and Immigration") OR (MH "Immigrants") OR (MH "Transients and Migrants") |

| Web of Science | |
| --- | --- |
| 10 | #9 AND #8 AND #3 AND #2 AND #1 |
| 9 | DOP=(2004-05-01)/2022-07-15) |
| 8 | #7 OR #6 OR #5 OR #4 |
| 7 | TS=(attitude* NEAR/3 health) |
| 6 | TS=(health NEAR/2 facilit*) |
| 5 | TS=(health NEAR/2 behavio*) |
| 4 | TS=(healthcare or “health-care” or care or “health service*” or medica* or NHS or “national health servic*” or “health belie*” or “health-seeking” |
| 3 | TS=(“United Kingdom” or UK or Britain or “Northern Ireland” or Wales or Scotland or England or London |
| 2 | TS=(migra* or emigra* or immigra* or foreign* or diaspor*) |
| 1 | TS=(“Eastern Europ*" OR "East Europ*" OR "Central Europ*" OR "Baltic Stat*" OR Bulgari* OR “Czech Republic” OR Czec* OR Croat* OR Estoni* OR Latvi* OR Lithuani* OR Hungar* OR Slovaki* OR Sloveni* OR Poland OR Polish OR Pole OR Poles OR Polska OR Bulgari* OR Roma* OR Roma |

| SCOPUS |
| --- |
| ( TITLE-ABS-KEY ( healthcare OR "health-care" OR "health service*" OR medical OR nhs OR "national health service*" OR care OR (health W/2 facilit*) OR (attitude W/3 health) OR (health W/2 behavio*) ) ) AND ( TITLE-ABS-KEY ( "United Kingdom" OR uk OR britain OR "Northern Ireland" OR wales OR scotland OR england OR london ) ) AND ( TITLE-ABS-KEY ( "Eastern Europe*" OR "East Europe*" OR "Central Europe*" OR "Czech Republic" OR czech* OR estonia* OR latvia* OR lithuania* OR hungar* OR slovaki* OR sloveni* OR poland OR polish OR pole OR poles OR polska OR bulgari* OR roma* OR roma OR croati*) ) AND ( TITLE-ABS-KEY ( migra* OR emigra* OR immigra* OR foreig* OR diaspor* ) ) PUBYEAR > 2003 AND ( LIMIT-TO ( LANGUAGE,"English" ) ) |

| Social Policy and Practice | |
| --- | --- |
| 1 | (migra* or immigr* or emigra* or foreign* or Diaspora*).mp |
| 2 | (Eastern Europe* or East Europe* or Central Europe* or Baltic State* Czech* or Czech Republic or Estonia* or Latvia* or Lithuania* or Hungar* or Slovaki* or Sloveni* or Poland or Poles or Pole or Polish or Polska or Bulgari* or Roma* or Roma or Croati*).mp |
| 3 | (United Kingdom or UK or England or Britain or London or Wales or Scotland or Northern Ireland).mp |
| 4 | ("health-care" or healthcare or health service* or medical or NHS or "national health service*" or care or "health-seeking" or health belief*).mp |
| 5 | (health adj2 facilit*).mp |
| 6 | (attitude* adj3 health).mp |
| 7 | (health adj2 behavio*).mp |
| 8 | 4 or 5 or 6 or 7 |
| 9 | 1 and 2 and 3 and 8 |
| 10 | limit 9 to yr="2004-2022" |

| Global Health | |
| --- | --- |
| 1 | migration/ or migrants/ or emigration/ |
| 2 | (migra* or emigra* or immigra* or foreign* or diaspora*).mp. [mp=abstract, title, original title, heading words, cabicodes words] |
| 3 | 1 or 2 |
| 4 | central europe/ or czech republic/ or hungary/ or poland/ or slovakia/ or slovenia/ or baltic states/ or estonia/ or latvia/ or lithuania/ or bulgaria/ or romania/ or croatia/ |
| 5 | (Eastern Europe* or East Europe* or Central Europe* or Baltic State* or Czech* or Czech Republic or Estonia* or Latvia* or Lithuania* or Hungar* or Slovaki* or Sloveni* or Poland or Poles or Pole or Polish or Polska or Bulgari* or Roma* or Roma or Croati*).mp |
| 6 | 4 or 5 |
| 7 | uk/ or great britain/ or northern ireland/ or england/ or "east midlands of england"/ or eastern england/ or northern england/ or south east england/ or south west england/ or "west midlands of england"/ or "yorkshire and lancashire"/ or wales/ or scotland/ or eastern scotland/ or northern scotland/ or "scottish highlands and islands"/ or west scotland/ |
| 8 | (United Kingdom or UK or Great Britain or Britain or England or Wales or Scotland or Northern Ireland).mp |
| 9 | 7 or 8 |
| 10 | health care/ or primary health care/ or tertiary health care/ or health services/ |
| 11 | (healthcare or "health-care" or health service* or medical or NHS or "national health service*" or care or "health-seeking" or health belief*).mp. |
| 12 | health beliefs/ or attitudes to health/ or health facilities/ |
| 13 | (health adj2 facilit*).mp. [mp=abstract, title, original title, heading words, cabicodes words] |
| 14 | (attitude* adj3 health).mp. [mp=abstract, title, original title, heading words, cabicodes words] |
| 15 | (health adj2 behavio*).mp. [mp=abstract, title, original title, heading words, cabicodes words] |
| 16 | 10 or 11 or 12 or 13 or 14 or 15 |
| 17 | 3 and 6 and 9 and 16 |
| 18 | limit 18 to yr="2004-2022" |

| Google Scholar |
| --- |
| “Central and Eastern European migrants” and “health” |

**Appendix 4: Codebook**

| **Themes** | **Subthemes** | **Codes** |
| --- | --- | --- |
| 1. Healthcare Bricolage/Using Healthcare in UK and/or in CoO | 1.1 Cultural Expectations of Medical Services | - 1. Bypass PHC to gain access to specialist care   2. Different medical policies (vaccines, antibiotics) |
|  |  | - 1. Can easily get medicines (especially antibiotics) in CoO, get medicine not available in the UK   2. Waiting times |
|  | 1.2 Trust/Distrust | 2.1 Distrust of NHS  2.2 Negative experiences  2.3 Want second opinion  2.4 Reassurance of diagnosis made in UK |
|  | 1.3 Barriers | 3.1 Documentation brought back difficult to translate |
|  |  | 3.2 Immediate results/copies  3.3 Precarious labour, difficult to take time off  3.4 Language  3.5 Using interpreter difficult/embarrassing |
|  | 1.4 Transnational Ties | 4.1Family in Poland that will help, can look after migrant has surgery/  4.2 Traveling to CoO to see family/friends, seek healthcare while there  4.3 Retain contact with health care professionals in CoO |
| 2. Using health services exclusively in the UK | 2.1 Trust/Distrust | 1.1 Distrust of Romanian healthcare system  1.2 Expectations of gratuities/bribes in CoO |
|  | 2.2 Financial Considerations | 2.1 Can’t afford to go back – travel costs, paying for healthcare in Poland |
|  | 2.3 Identity | 3.1 Pay taxes in the UK, living in the UK – why use healthcare elsewhere? |

**Appendix 5: Results of individual sources**

| Author, Year, Title | CEE Nationality/Population | Years in UK | UK Location | Cultural Expectations of Medical Services | Trust/Distrust | Barriers | Transnational Ties | Findings |
| --- | --- | --- | --- | --- | --- | --- | --- | --- |
| Horsfall (2020). "Medical Tourism from the UK to Poland: How the Market Masks Migration" | Polish | N/A | Across all UK |  |  |  |  |  |
| Bell et. al (2019). "I don't think anybody explained to me how it works': qualitative study exploring vaccination and primary health service access and uptake amongst Polish and Romanian communities in England" | 20 Polish, 10 Romanian 27/30 mothers or pregnant women | Average time was 11 years for Polish, 9 years for Romanian | Lincolnshire, Berkshire, London as well as out of area | Bypass PHC to gain access to specialist care Concerns that nurses administer vaccines in UK vs doctors in Poland Concerns that children not tested before vaccine administered/no check up | Lack of confidence in GPs | Knowledge of health care system Language | Traveling to CoO for leisure -> received healthcare there | No documentation brought back Documentation brought back difficult to translate |
| Gorman (2019). "A qualitative study of vaccination behaviour amongst female Polish migrants in Edinburgh, Scotland" | Polish, n=13, all women Ages ranged between 20-70 | Varied between 2-15 years | Edinburgh, Scotland | Additional vaccines not offered in UK GPs not examining/doing check-up on child prior to vaccination | Distrust of GPs | Language |  |  |
| Gorman et. al (2017). "Influences on Polish migrants' breast screening uptake in Lothian, Scotland" | Polish women n=11 Ages 50-65, avg age 58 | Years living in Lothian Range from 2-15 Avg=8 | Lothian, Scotland | Bypass PHC to gain access to specialist care | Second opinion/reassurance | Difficult to take time off work for medical appointments Language | Traveling to CoO for leisure -> received healthcare there | Satisfaction with hospital care |
| Jackowska et. al (2011). "Cervical screening among migrant women: a qualitative study of Polish, Slovak and Romanian women in London, UK" | n=52, all women Polish = 29 Slovak.= 12 Romanian = 11 Ages between 20-50+ | 41 women ranging between 1-9 yrs 10 women ranging from 10-20+ | London |  | Second opinion/reassurance | Difficult to take time off work for medical appointments | Visiting home, so see HCP while there | Romanian women distrust of healthcare in Romania, so used NHS in England Dual usage for Polish women |
| Main (2016). "Biomedical practices from a patient perspective. Experiences of Polish female migrants in Barcelona, Berlin and London" | Polish women n=2 (8 overall, 2 in London) Ages between 25-40 | At least 1 yr, majority at least 5 years | London | Underwhelmed by pregancy healthcare offered Getting medicine not available in UK/different medications | Distrust of NHS |  |  | Pregnancy in Poland "overmedicalized", women were happy with the patient-centered care received in London |
| Moreh et. al (2022). "Transnational Healthcare Preferences Among EU Nationals in the UK: A Qualitative Assessment" | Polish = 277 Hungarian = 45 Romanian = 39 Other non CEE nationalities included, Polish constituted 54.2% | No info | All four nations | the paracetamol service - easier to gain access to antibiotics | Second opinion/reassurance | Language | Family support in Poland to look after having surgery |  |
| Nelson et. al (2021). "Experiences of cervical screening participation and non-participation in women from minority ethnic populations in Scotland" | Polish (9), Romanian (1) women Ages varied 30-59 | Varied 5-20+ years | Scotland | Bypass PHC to gain access to specialist care |  |  |  |  |
| Osipovic (2013). "If I get ill, It’s onto the plane, and off to Poland. Use of health care services by polish migrants in London." | Polish, n=62 33 female, 29 male Ages varied 22-70, but 62% 22-34 | Varied 1-5+  50% between 2-5 years | London |  | Distrust of NHS Second opinion/reassurance | Language, lack of knowledge about NHS More cost effective to get healthcare abroad instead of taking time off in UK | Traveling to CoO for leisure -> received healthcare there |  |
| Sime (2014). "I think that Polish doctors are better': newly arrived migrant children and their parents' experiences and views of health services in Scotland" | Bulgarian, Czech, Hungarian, Lithuanian, Polish, Romanian, Slovak 57 children in focus-groups, 23 in-depth family case studies Mostly Polish from both | No info | Scotland | the paracetamol service - easier to gain access to antibiotics/other medications Bypass GP/waiting times to access specialist care | Second opinion/reassurance |  | Traveling to CoO for leisure -> received healthcare there | "human-centered" approach, children felt involved in their healthcare in Scotland |
| Troccoli et. al (2022) "Diagnostic testing: therapeutic mobilities, social fields, and medical encounters in the transnational healthcare practices of Polish migrants in the UK" | Polish, n=32 Female (n=22), Male (n=10) Ages between 20-69 | Varies 1-16, 78% between 5-14 yrs | England | Immediate results/copies in Poland | Second opinion/reassurance |  |  |  |
| Patel et. al (2020). "Awareness of and attitudes towards cervical cancer prevention among migrant Eastern European women in England" | Quantitative = Bulgarian(1), Czech (7), Hungarian (4), Latvian (6), Lithuanian (4), Polish (39), Romanian(4), Slovakian (17)  Qualitative = 20 one-to-one with EE women (no breakdown), one focus group with 6 women from Czech + Slovakian backgrounds Age range = 24-55, median of 31 | No info | England - Midlands | Bypass PHC to gain access to specialist care Not comfortable with nurses doing smear tests/thought it should be a specialist | Second opinion/reassurance, more trust in "at home" doctors | Language |  | Believed 3 year screening intervals were cost saving measures |
| Troccoli et. al (2021) "Transnational healthcare as process: multiplicity and directionality in the engagements with healthcare among Polish migrants in the UK" | Polish females and males Quantitative, n=482 Qualitative, n=32 | Inconclusive | Not specified/UK | Immediate results/copies in Poland Frustraton with GPs Bypass PHC to gain access to specialist care Different criteria for diagnosing | Second opinion/reassurance Distrust of NHS |  | Visiting CoO for non-health related matters, seek HCP while there |  |
| Main (2014). "Medical Travels of Polish Female Migrants in Europe" | Polish women n=18 (98 overall, 18 in London) Ages varied 22-65 | At least 1 yr, majority at least 5 years | London | Bypass PHC to gain access to specialist care Used to specialist care in Poland - different in the UK (for gynaecology and pediatrics) | Distrust of NHS Second opinion/reassurance |  | Traveling to CoO for leisure --> received healthcare there |  |
| Guma (2018). "Exploring Potentialities of (Health)Care in Glasgow and Beyond: Negotiations of Social Security Among Czech- and Slovak-Speaking Migrants" | 3 Czech, 6 Slovak speakers. Among them 2 identified as Roma 5 women, 4 men aged between 30-54 | No info | Glasgow, Scotland | Bypass PHC to gain access to specialist care "the paracetamol service" - easier to gain access to antibiotics Immediate results | Distrust of GPs |  | Traveling to CoO for leisure -> received healthcare there | Satisfaction with hospital care |
| Healthwatch Reading (2014). How the recent migrant Polish community are accessing healthcare services, with a focus on primary and urgent care services | Polish women and men (n=34) Ages between 20-51+ | Majority resident for at least 3 yrs <1 year, 1-10+ years | Reading, England | Bypass PHC to gain access to specialist care "the paracetamol service"- easier to gain access to antibiotics |  | Language - written and spoken | Traveling to CoO -> receieved healthcare there |  |
